# Expression of junctional proteins in choroid plexus epithelial cell lines: a comparative study

**DOI:** 10.1186/1743-8454-4-11

**Published:** 2007-12-27

**Authors:** Joanna Szmydynger-Chodobska, Crissey L Pascale, Andrew N Pfeffer, Cassaundra Coulter, Adam Chodobski

**Affiliations:** 1 Department of Clinical Neurosciences, The Warren Alpert Medical School of Brown University, Providence, RI 02903, USA

## Abstract

**Background:**

There is an increasing interest in using choroid plexus (CP) epithelial cell lines to study the properties of the blood-cerebrospinal fluid barrier (BCSFB). Currently, there are three major CP-derived cell lines available. Z310 and TR-CSFB3, two immortalized cell lines carrying the simian virus 40 large T-antigen gene, were derived from rat CP epithelium, whereas the CPC-2 cell line was derived from human CP carcinoma. Although these cell lines have previously been used in various functional studies, the expression of adherens junction (AJ) and tight junction (TJ) proteins in these epithelial cells has not been systematically studied. Accordingly, in the present study, we sought to characterize the expression of these junctional proteins in these three cell lines.

**Methods:**

The cells were grown in six-well cell culture plates. Reverse-transcriptase polymerase chain reaction, Western blotting, and immunocytochemistry were used to characterize the expression of AJ and TJ proteins in the CP cell lines.

**Results:**

Z310 and TR-CSFB3 cells expressed a TJ protein, occludin, and its cytosolic binding partner, zonula occludens 1, as well as an AJ protein, E-cadherin, and β-catenin, a cytoplasmic protein that interacts with E-cadherin. However, the expression of occludin and E-cadherin in TR-CSFB3 cells at both the mRNA and protein level was weaker than that found in Z301 cells. The immunocytochemical analysis also demonstrated that the staining pattern for these junctional proteins in TR-CSFB3 cells was discontinuous and the staining intensity was weaker than that observed in Z310 cells. The message for claudin 1 and claudin 2 was expressed at low levels in TR-CSFB3 cells and these cells were weakly immunopositive for claudin 1. In comparison, the message for these TJ proteins could not be detected in Z310 cells. CPC-2 cells expressed occludin, which was localized to areas of cell-cell contact, but the staining pattern for this TJ protein was found to be variable and irregular. Although CPC-2 cells expressed mRNA for claudin 1, claudin 2, and claudin 11, only claudin 1 was expressed at the protein level and it was localized to the nuclei rather than to areas of cell-cell contact. An AJ protein, E-cadherin, was also found to be mislocalized in CPC-2 cells, even though its cytosolic binding partner, β-catenin, was restricted to areas of cell-cell contact, as in normal CP.

**Conclusion:**

The three CP cell lines analyzed in this study vary considerably with regard to the expression of AJ and TJ proteins, which is likely reflected by different barrier properties of these *in vitro *models of BCSFB.

## Background

There is an increasing interest in using choroid plexus (CP) epithelial cell lines to study the properties of the blood-cerebrospinal fluid (CSF) barrier (BCSFB). The advantage of using the CP cell lines is not only the lower cost associated with conducting the experiments, but also the relative ease of growing and genetically manipulating these cells compared to primary cultures of choroidal epithelium. Currently, there are three major CP-derived cell lines available to study the properties of the BCSFB. The Z310 immortalized cell line was derived from primary cultures of rat CP epithelium transfected with a plasmid carrying the simian virus 40 (SV40) large T-antigen gene [1]. These cells display polygonal morphology typical of choroidal epithelial cells and form monolayers with the transepithelial electrical resistance (TEER) varying between ~60 and 150–200 Ω·cm^2 ^[1,2], which is comparable with the TEER values found for primary cultures of CP epithelium from the rat [2,3]. Zheng and collaborators have demonstrated that Z310 cells produce transthyretin (TTR), a marker for the choroidal epithelium, and express a number of transporters, including members of the family of ATP-binding cassette transporters, ABCB1 (P-glycoprotein/multidrug resistance 1) and ABCC1 (multiple drug resistance protein 1), organic cation transporter 1, and several metal transporters (the members of the solute carrier superfamily of transporters), such as SLC11A2 (divalent metal transporter 1), SLC30A1 (zinc transporter 1), and SLC40A1 (metal transporting protein 1), as well as the copper-transporting ATPase, ATP7A [1,4]. The organic anion transporter 3 was also found to be expressed in the Z310 line, albeit at much lower levels than those observed in the CP.

A slightly different approach has been chosen by Terasaki and colleagues to establish five immortalized cell lines of CP epithelium, TR-CSFB1-5. These cell lines were derived from cultures of choroidal epithelial cells harvested from transgenic rats harboring a temperature-sensitive SV40 large T-antigen gene [5]. When grown at the permissive temperature of 33°C, these cells form monolayers with polygonal epithelial morphology and TEER of ~50 Ω·cm^2^. Among TR-CSFB lines, the TR-CSFB3 line was characterized with greater detail. Similar to Z310 cells, the TR-CSFB3 line synthesizes TTR and expresses several transporters, including ABCA1 and 4, ABCB1, ABCC1, and ABCG1 and 2, which belong to the family of ATP-binding cassette transporters [5-9]. Organic anion transporting polypeptide 3 was also reported to be expressed in TR-CSFB3 cells, but the levels of expression of this transporter were much lower than those found in the CP. This group has also conducted biochemical studies on TR-CSFB3 cells to show that they have the capability to actively transport L-proline and L-glutamate [5].

The CPC-2 cell line was derived from human CP carcinoma [10]. Although CPC-2 cells have not yet been characterized with regard to barrier function, their polypeptide secretory activity, an important feature of choroidal epithelium [11], has been studied. CPC-2 cells have been shown to produce endothelin 1 (ET-1) and adrenomedullin (ADM) [12,13]. The exposure of CPC-2 cells to tumor necrosis factor-α, interleukin 1β, or a combination of these two cytokines, resulted in the upregulation of message for ET-1 and ADM as well as increased secretion of these proteins into the culture media, suggesting that the CPC-2 line represents a valuable *in vitro *model for studying the polypeptide secretory function of the CP.

**Figure 1:**
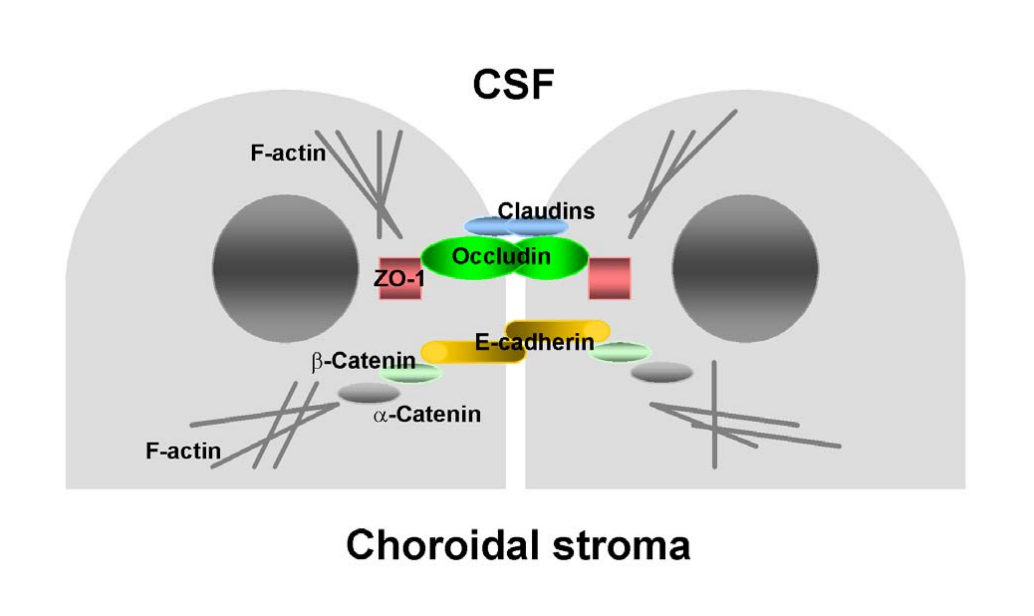
Schematic representation of AJ and TJ complexes in choroidal epithelial cells. AJs are formed by E-cadherin, a single pass transmembrane protein that exhibits Ca^2+^-dependent homophilic interactions. The intracellular domain of E-cadherin binds to β-catenin, which binds to α-catenin, which in turn interacts with actin, and this catenin-mediated anchorage of E-cadherin to the actin cytoskeleton is required for strong cell-cell adhesion. Occludin and the members of the family of claudin proteins are the major constituents of TJs. Occludin and claudins are connected to the actin filaments through cytoplasmic adaptor protein, ZO-1.

The integrity, paracellular permeability, and polarization of epithelial monolayers depend on the expression of adherens junction (AJ) and tight junction (TJ) proteins. Figure 1 shows a schematic representation of AJ and TJ complexes in choroidal epithelial cells. AJs are formed by E-cadherin, a single pass transmembrane protein that exhibits Ca^2+^-dependent homophilic interactions [14]. The intracellular domain of E-cadherin binds to β-catenin, which binds to α-catenin, which in turn interacts with actin, and this catenin-mediated anchorage of E-cadherin to the actin cytoskeleton is required for strong cell-cell adhesion [14]. E-cadherin, as well as α-catenin and β-catenin, are expressed in choroidal epithelium [15]. The formation of AJs plays an important role in generating the polarized distribution of plasma membrane proteins [16] and initiates the formation of other cell-cell junctional complexes, such as TJs and desmosomes [17-19]. TJs form a barrier preventing solutes from moving along the paracellular pathways. They also restrict the diffusion of integral membrane proteins and lipids between the apical and basolateral plasma membrane domains [20]. Occludin and the members of the family of claudin proteins, belonging to the superfamily of tetraspanins (proteins containing four transmembrane domains), are the major constituents of TJs [20]. Occludin, as well as claudin 1, claudin 2, and claudin 11, have been reported to be expressed in choroidal epithelium [21,22]. Occludin and claudins are connected to the actin filaments through cytoplasmic adaptor proteins, zonula occludens 1–3 (ZO-1-3), members of the membrane-associated guanylate kinase family of proteins [20]. Interestingly, ZO-1 not only binds to occludin and claudins, but is also able to interact with α-catenin [23]. However, in epithelial cells bearing well-developed TJs, ZO-1 does not co-localize with AJs [24].

Although the Z310 cells have been partially characterized with regard to the expression of TJ proteins [2,4], the expression of AJ proteins in this cell line has not been analyzed. The expression of junctional proteins in other CP epithelial cell lines, namely TR-CSFB3 and CPC-2, has not been studied. Accordingly, in the present study, we sought to characterize the expression of AJ and TJ proteins in these three cell lines.

## Methods

### Reagents and antibodies

ThermoScript RNase H^- ^reverse transcriptase, RNase inhibitor, RNaseOut, and Platinum *Taq *DNA polymerase were obtained from Invitrogen (Carlsbad, CA, USA). Polyclonal rabbit antibodies to occludin, claudin 1, claudin 2, and ZO-1 were purchased from Zymed Labs (South San Francisco, CA, USA), whereas monoclonal mouse antibodies to α-catenin, β-catenin, and E-cadherin were obtained from BD-Transduction Labs (Lexington, KY, USA). For detection on Western blots, we used horseradish peroxidase-conjugated anti-rabbit (from donkey) and anti-mouse (from sheep) IgGs (Amersham Biosciences, Piscataway, NJ, USA). Secondary antibodies for immunocytochemistry were purchased from Molecular Probes (Eugene, OR, USA). These were goat anti-rabbit and anti-mouse IgGs conjugated with Alexa 488. Normal goat serum was obtained from Jackson Immunoresearch Labs (West Grove, PA, USA) and Vectashield mounting medium for fluorescence microscopy was from Vector Labs (Burlingame, CA, USA).

### Cell lines

The Z310 cell line was kindly provided by Dr. Wei Zheng (Purdue University, West Lafayette, IN, USA). Low-glucose Dulbecco's modified Eagle medium (DMEM) supplemented with penicillin (100 U/ml), streptomycin (100 μg/ml), gentamicin (40 μg/ml), and 10% fetal bovine serum (FBS) was used to culture Z310 cells. They were grown to confluence in untreated six-well cell culture plates at 37°C in a humidified atmosphere of 5% CO_2_/95% air. The TR-CSFB3 cell line was a kind gift of Dr. Tetsuya Terasaki (Graduate School of Pharmaceutical Sciences, Tohoku University, Sendai, Japan). TR-CSFB3 cells were cultured in collagen I-coated plates in high-glucose DMEM supplemented with penicillin (100 U/ml), streptomycin (100 μg/ml), and 10% FBS. They were initially grown at 33°C in 5% CO_2_/95% air and after reaching confluence were maintained at 37°C. The CPC-2 cell line was provided by Dr. Toshio Kudo (Cell Resource Center for Biomedical Research, Institute of Development, Aging and Cancer, Tohoku University, Sendai, Japan). CPC-2 cells were cultured in high-glucose DMEM supplemented with penicillin (100 U/ml), streptomycin (100 μg/ml), and 10% FBS. They were grown to confluence in collagen I-coated plates at 37°C in 5% CO_2_/95% air. The cell culture media, FBS, and antibiotics were obtained from Invitrogen.

### Choroid plexus tissues

Samples of the lateral ventricle CP were from Sprague-Dawley rats (Charles River Breeding Labs, Wilmington, MA, USA). Rats were euthanized with intraperitoneal pentobarbital sodium (150 mg/kg), brains were removed, and CPs subsequently excised. These procedures were in accordance with the guidelines of the Animal Care and Use Committee of Rhode Island Hospital and conformed to international guidelines on the ethical use of animals. All efforts were made to minimize the number of animals used and their suffering. Samples of human choroidal tissue were provided by Dr. Edward G. Stopa (Brown University Medical School, Providence, RI, USA). These were specimens of the lateral ventricle CP from one male patient without neurological disease. They were collected at autopsy performed within 10 h after death. Immediately after collection, the specimens were processed for RNA and protein extraction as described below.

### Reverse-transcriptase polymerase chain reaction (RT-PCR)

Total RNA was isolated using NucleoSpin RNA II kit (Macherey-Nagel, Düren, Germany). First-strand cDNAs were synthesized using 50 pmol of oligo(dT)_20 _primer and 15 U of ThermoScript reverse transcriptase. Forty units of RNase inhibitor, RNaseOut, were included in the reverse transcription reactions. For each 20-μl reaction, 1 μg of total RNA was used and the reaction was carried out for 1 h at 50°C.

The sequences of primers and the predicted sizes of PCR products are given in Table 1. The 25-μl PCR reaction mixtures contained 0.2 mM mixed dNTPs, 0.2 μM each primer, 1.5 mM MgCl_2_, 1 U Platinum *Taq *DNA polymerase, and 1/40 of the reverse transcription reaction product. The PCR reaction mixtures were heated to 95°C for 2 min and then were subjected to 30–35 cycles of denaturation (94°C, 15 s), annealing (57°C, 15 s), and extension (72°C, 15 s). A final extension was carried out at 72°C for 5 min. The PCR products (10 μl of each PCR reaction) were separated on a 1.8% agarose gel and were visualized by ethidium bromide staining under UV light. Control experiments were also performed in which reverse transcriptase was omitted from the reverse transcription reactions and 1/40 of the product of each reaction was used for PCR. Under these conditions, no bands were seen on agarose gels for any of the genes studied with 35 cycles of amplification (data not shown).

**Table 1:** The sequences of primers and the predicted sizes of PCR products.

Species	Gene	Primer	Sequence	Predicted size of PCR product (base pairs)
Rat	Occludin	F	5'-GGAACACATTTATGATGAACAGC-3'	235
		R	5'-CACGGACAAGGTCAGAGGAA-3'	
	Claudin 1	F	5'-AAAGATGTGGATGGCTGTCA-3'	189
		R	5'-AGGAGGCAGAGGGAGGC-3'	
	Claudin 2	F	5'-AGGGTTTCCTTAGGGACAATAA-3'	196
		R	5'-TAAAGTATCTGGTAGGGTTGCC-3'	
	Claudin 11	F	5'-CTCATCCTCCCTGGTTACG-3'	175
		R	5'-GCAGAATAAGGAGCACCCC-3'	
	E-cadherin	F	5'-AAGAAGACCAGGACTTTGATTTG-3'	169
		R	5'-GCTGCCTTCAGGTTTTCATC-3'	
				
Human	Occludin	F	5'-AGGAACACATTTATGATGAGCAG-3'	242
		R	5'-GAAGTCATCCACAGGCGAA-3'	
	Claudin 1	F	5'-GAAGATGAGGATGGCTGTCA-3'	142
		R	5'-AAATTCGTACCTGGCATTGA-3'	
	Claudin 2	F	5'-ACCATTCCTTGACGGTGTCTA-3'	121
		R	5'-GCTGATTTTCCATTACGCCT-3'	
	Claudin 11	F	5'-TCATCCTGCCGGGCTAC-3'	174
		R	5'-GCAGAATGAGCAAAACACCA-3'	
	E-cadherin	F	5'-CCTGCCAATCCCGATGA-3'	199
		R	5'-TGCCCCATTCGTTCAAGTA-3'	

*F = forward; R = reverse.

### Western blotting

The cells were scraped into isotonic lysis buffer containing 150 mM NaCl, 50 mM Tris-HCl, pH 7.4, 2 mM EDTA, 1% SDS, 0.5% deoxycholate, 1% Triton X-100 (TX-100), 1 mM PMSF, 1 mM benzamidine, 100 U/ml aprotinin, 20 μg/ml antipain, 20 μg/ml leupeptin, and 1 μg/ml pepstatin. Proteins were separated via SDS-polyacrylamide gel electrophoresis (8% for occludin, 14% for claudin 1 and claudin 2, and 6% for E-cadherin) under reducing conditions and then were transferred onto 0.2-μm supported nitrocellulose membranes (Bio-Rad Labs, Hercules, CA, USA). After blocking with 5% non-fat milk for 1 h at room temperature, the membranes were incubated with primary antibodies overnight at 4°C. The following concentrations of primary antibodies were used: 0.125 μg/ml for anti-occludin antibody and 2.5 μg/ml for anti-claudin 1, anti-claudin 2, and anti-E-cadherin antibodies. Membranes were subsequently incubated with horseradish peroxidase-conjugated anti-rabbit (diluted 1:2,000) or anti-mouse (diluted 1:2,500) IgG for 1 h at room temperature. For detection, ECL chemiluminescence system (Amersham Biosciences) was used.

### Immunocytochemistry

For immunocytochemistry, the cells were grown on glass cover slips. For TR-CSFB3 and CPC-2 cells, the cover slips were precoated with collagen I. Cells were fixed with 4% paraformaldehyde in 0.01 M phosphate-buffered saline (PBS), pH 7.4 for 10 min at room temperature, followed by two 5-min washes in PBS. The immunocytochemical procedures were performed at room temperature, except for the incubation with primary antibodies that was completed at 4°C. All incubations were performed in PBS containing 0.25% bovine serum albumin (BSA) and 0.25% TX-100. For washes, PBS containing 0.1% BSA and 0.1% TX-100 was used. To minimize non-specific staining, the cells were incubated for 30 min with 10% normal goat serum. Four percent of normal goat serum was also added when the cells were incubated with primary and secondary antibodies. After the initial blocking step, the cells were incubated overnight with primary antibodies. The following concentrations of primary antibodies were used: 0.25 μg/ml for anti-occludin antibody, 0.5 μg/ml for anti-ZO-1 and anti-E-cadherin antibodies, and 2.5 μg/ml for anti-claudin 1, anti-claudin 2, anti-α-catenin, and anti-β-catenin antibodies. Six 10-min washes were then performed and the cells were incubated for 1 h with secondary antibodies at a concentration of 2 μg/ml. After four 10-min washes, the cells were mounted with Vectashield mounting medium. The cells were viewed with a fluorescence microscope (Olympus, Model BH2-RFCA).

## Results

Results are summarized in Table 2.

**Table 2:** Summary of results.

**Junctional protein**	**Z310 cells**			**TR-CSFB3 cells**			**CPC-2 cells**		
	
	mRNA RT-PCR	Protein WB	Protein ICC	MRNA RT-PCR	Protein WB	Protein ICC	mRNA RT-PCR	Protein WB	Protein ICC
Occludin	+	+	+	+	+	+	+	+	+
Claudin 1	-	-	-	+	-	+	+	+	+*
Claudin 2	-	-	-	+	-	-	+	-	-
Claudin 11	-	ND	ND	-	ND	ND	+	ND	ND
ZO-1	ND	ND	+	ND	ND	+	ND	ND	-
E-cadherin	+	+	+	+	+	+	+	-	+*
α-Catenin	ND	ND	+	ND	ND	-	ND	ND	-
β-Catenin	ND	ND	+	ND	ND	+	ND	ND	+

* = mislocalized; WB = Western blotting; ICC = immunocytochemistry; ND = not determined.

### RT-PCR analysis

**Figure 2:**
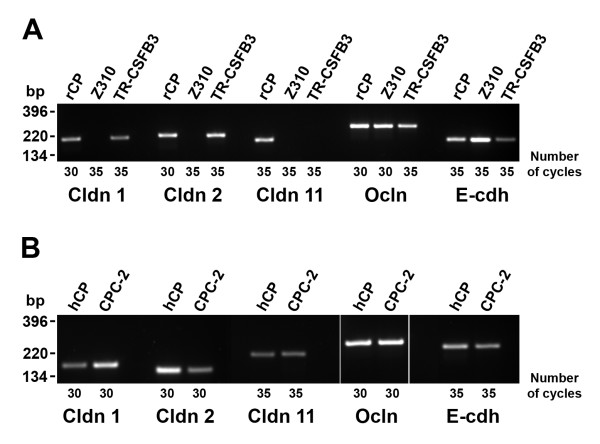
RT-PCR analysis of expression of junctional proteins in CP cell lines. A. Expression of TJ and AJ proteins in Z310 and TR-CSFB3 cells, two immortalized cell lines derived from rat CP epithelium. B. Expression of TJ and AJ proteins in the CPC-2 cell line derived from human CP carcinoma. The number of cycles of amplification is shown for each lane. Cldn 1, Cldn 2, Cldn 11, Ocln, and E-cdh are claudin 1, claudin 2, claudin 11, occludin, and E-cadherin, respectively. rCP and hCP are rat and human CP tissues used as a positive control.

The expression levels of TJ proteins, occludin and claudins, and an AJ protein, E-cadherin, varied considerably among the studied cell lines. Based on previous observations that claudin 1, claudin 2, and claudin 11 are expressed in rodent CP [21,22], we focused our analysis on these three members of the claudin family. None of these TJ proteins were found to be expressed in Z310 cells derived from rat CP (Fig. 2A). In another epithelial cell line derived from rat CP, the TR-CSFB3 line, claudin 1 and claudin 2 were weakly expressed, but the message for claudin 11 was not detected (Fig. 2A). In comparison, in human CPC-2 cells, all three members of the claudin family were expressed (Fig. 2B). Unlike claudins, occludin was expressed in all three cell lines (Figs. 2A and 2B). Similarly, the message for E-cadherin was expressed in rat and human CP cell lines (Figs. 2A and 2B).

### Western blotting

**Figure 3:**
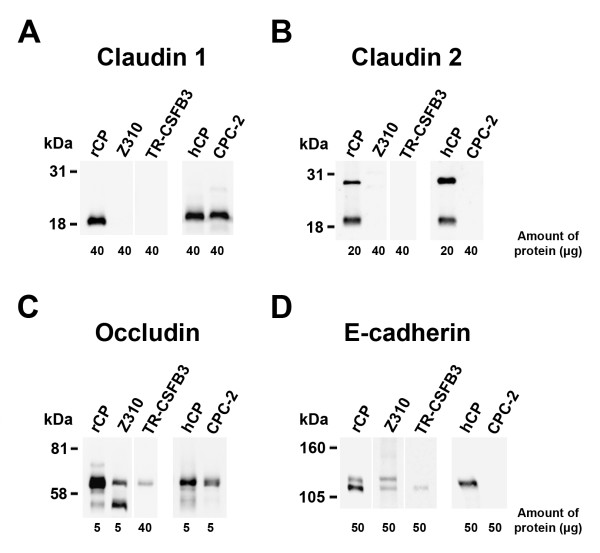
Western blot analysis of expression of junctional proteins in CP cell lines. A, B, and C. Expression of TJ proteins, claudin 1, claudin 2, and occludin. D. Expression of AJ protein, E-cadherin. Z310 and TR-CSFB3 cell lines were derived from rat CP epithelium, whereas the CPC-2 cell line was derived from human CP carcinoma. The amount of protein loaded is shown for each lane. rCP and hCP are rat and human CP tissues used as a positive control.

The expression of claudins at the protein level did not consistently reflect their expression at the mRNA level. Although the message for claudin 1 and claudin 2 was found in TR-CSFB3 cells, these two members of the claudin family could not be detected in protein extracts from these cells on immunoblots (Figs. 3A and 3B). Similarly, claudin 2 was not detected in protein extracts from CPC-2 cells even though the message for this TJ protein was found in this cell line (Fig. 3B). In comparison, claudin 1 was found to be expressed in CPC-2 cells at both the mRNA and protein level (Fig. 3A). Neither claudin 1 nor claudin 2 could be detected in protein extracts from Z310 cells (Figs. 3A and 3B), which was consistent with the data obtained from mRNA analysis. As expected, claudin 2 was detected as a ~22-kDa protein in the rat and human choroidal tissues; however, an additional band with higher molecular weight was also consistently observed on immunoblots, which may have represented the glycosylated form of claudin 2. Occludin was detected as a ~65-kDa protein; however, additional bands with both lower and higher molecular weight and lower intensity (except for Z310 cells) were found on immunoblots (Fig. 3C). These multiple bands are likely to represent various isoforms of occludin resulting from alternative splicing events [25]. In TR-CSFB3 cells, occludin was found to be expressed at a lower level compared to other two lines (Fig. 3C). E-cadherin was detected on immunoblots as a ~120-kDa protein (Fig. 3D). Interestingly, an additional band of ~135 kDa was also found in protein extracts from rat choroidal tissue and Z310 cells. This additional band may represent a precursor of E-cadherin [26]. E-cadherin was only weakly expressed in TR-CSFB3 cells and could not be detected in protein extracts from CPC-2 line (Fig. 3D).

### Immunocytochemistry

#### Z310 cells

**Figure 4:**
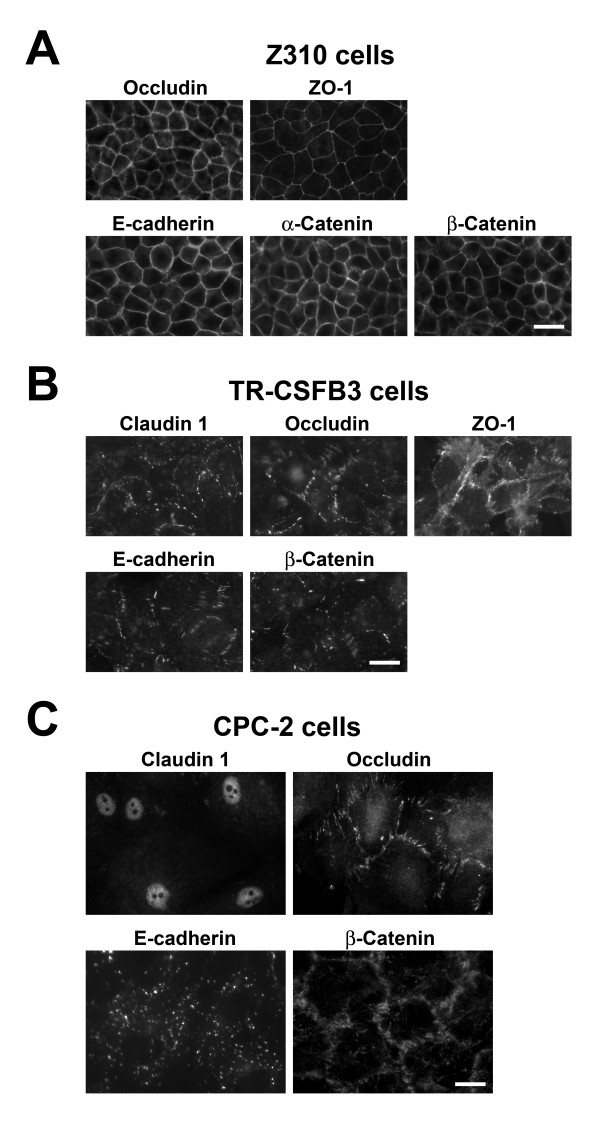
Immunocytochemical analysis of expression of junctional proteins in CP cell lines. A and B. Z310 and TR-CSFB3 cell lines derived from rat CP epithelium. C. CPC-2 cell line derived from human CP carcinoma. Scale bars = 10 μm.

Z310 cells had cobblestone-like morphology typical of CP epithelium. As expected, based on RT-PCR analysis and Western blotting, the Z310 line expressed occludin and E-cadherin, and these proteins were found to be localized to areas of cell-cell contact in confluent monolayers with a continuous pattern of immunostaining (Fig. 4A). We also showed that Z310 cells express ZO-1, a cytoplasmic adaptor protein binding to claudins and occludin [23], and α-catenin and β-catenin, the E-cadherin interacting proteins [14]. Similar to occludin and E-cadherin, ZO-1 and catenins had a continuous pattern of immunostaining and were localized to areas of cell-cell contact (Fig. 4A). Consistent with the RT-PCR results, the Z310 cells did not stain for claudin 1 and claudin 2 (data not shown).

#### TR-CSFB3 cells

TR-CSFB3 cells formed less regular monolayers compared to Z310 cells. Similar to Z310 cells, they expressed occludin and E-cadherin, which were localized to areas of cell-cell contact. However, the pattern of this immunostaining was frequently discontinuous and the intensity of staining was weaker than that found in Z310 cells (Fig. 4B). Interestingly, the TR-CSFB3 cells were also found to weakly stain for claudin 1 (Fig. 4B), even though this TJ protein could not be detected in protein extracts from these cells by Western blotting. This discrepancy reflects the higher sensitivity of immunofluorescence technique compared to immunoblotting. TR-CSFB3 cells did not stain for claudin 2 (data not shown), suggesting that the message for this TJ protein is inefficiently translated in these cells. Similar to Z310 cells, the TR-CSFB3 line was found to be immunopositive for ZO-1 and β-catenin (Fig. 4B), but it did not express α-catenin (data not shown). Although ZO-1 and β-catenin were found to be localized to areas of cell-cell contact, the pattern of immunostaining for these two proteins was discontinuous and the intensity of staining was weaker than that found in Z310 cells (Fig. 4B).

#### CPC-2 cells

Similar to TR-CSFB3 cells, the CPC-2 line formed less regular monolayers compared to Z310 cells. In addition, the CPC-2 cells were larger in size than Z310 and TR-CSFB3 cells. Although they expressed occludin, which was localized to areas of cell-cell contact, the staining pattern for this TJ protein was variable and irregular (Fig. 4C). As expected, based on both RT-PCR and Western blotting, the CPC-2 cells expressed claudin 1; however, this TJ protein was found to be restricted to the nuclei of these epithelial cells (Fig. 4C). CPC-2 cells did not stain for claudin 2 or ZO-1 (data not shown). E-cadherin was expressed in CPC-2 line, but the immunoreactive product for this AJ protein was not localized to areas of cell-cell contact (Fig. 4C). Interestingly, β-catenin, a cytosolic protein that binds to E-cadherin [14], was localized to areas of cell-cell contact; however, similar to occludin, the staining pattern for β-catenin was variable and irregular (Fig. 4C). Another member of AJ complex, α-catenin, was not detected in CPC-2 cells (data not shown).

## Discussion

In the present study, the junctional proteins in two immortalized epithelial cell lines derived from rat CP were analyzed. Although the same oncogene (SV40 large T-antigen) was used to generate the Z310 and TR-CSFB3 lines, these two CP cell lines differ with regard to expression of junctional proteins. Both cell lines were found to express a TJ protein, occludin, and its cytosolic binding partner, ZO-1, as well as an AJ protein, E-cadherin, and β-catenin, a cytoplasmic protein that interacts with E-cadherin. However, the expression of occludin and E-cadherin in TR-CSFB3 cells at both the mRNA and protein level was weaker than that found in Z301 cells. The immunocytochemical analysis also demonstrated that the staining pattern for these junctional proteins in TR-CSFB3 cells was discontinuous and the staining intensity was weaker than that observed in Z310 cells. Interestingly, the mRNA for claudin 1 and claudin 2 was expressed at low levels in TR-CSFB3 cells and these cells were only weakly immunopositive for claudin 1. In comparison, in Z310 cells, the message for these TJ proteins could not be detected, which was consistent with the results of a previous study by Shi and Zheng [2]. Our findings are in line with observations that the expression of TJ and AJ proteins in immortalized epithelial cells carrying the SV40 large T-antigen gene can vary considerably. For example, gastric and colonic epithelial cell lines have been reported to express high levels of ZO-1 and E-cadherin [27,28], whereas introduction of the SV40 large T-antigen gene to uterine epithelial cells resulted in the loss of these junctional proteins [29].

The CPC-2 cell line was derived from human CP carcinoma [10]. These epithelial cells express occludin, which is localized to areas of cell-cell contact, but the staining pattern for this TJ protein was found to be variable and irregular. Although the CPC-2 cells express mRNA for claudin 1, claudin 2, and claudin 11, only claudin 1 was found to be expressed at the protein level and it was localized to the nuclei rather than to areas of cell-cell contact, as in normal CP [21,22]. An AJ protein, E-cadherin, was also found to be mislocalized in CPC-2 cells, and α-catenin, a component of the AJ complex, could not be detected. These results are consistent with frequently observed dysregulation of AJ complexes occurring in carcinoma cells [30] and are in line with previously reported disruption of TJs caused by oncogenes [31]. Similar to our findings, a recent study has demonstrated the nuclear localization of claudin 1 in a number of carcinoma cells [32]. Unexpectedly, overexpression of claudin 1 in a colon carcinoma line expressing low levels of this TJ protein and having epithelioid morphology resulted in the redistribution of E-cadherin from areas of cell-cell contact and epithelial-mesenchymal transition of these cells. These latter findings suggest that, under certain conditions, claudin 1 may act to weaken the epithelial barrier.

Interestingly, β-catenin, a cytosolic protein, was found to be restricted to areas of cell-cell contact in CPC-2 cells, even though E-cadherin, an integral membrane protein and the binding partner for β-catenin, had a different cellular distribution. These observations are similar to those previously reported for a human breast epithelial cell line transfected with the Ha-Ras protooncogene, where β-catenin and E-cadherin did not co-localize in areas of cell-cell contact [33]. These findings raise an important question as to which membrane-associated protein β-catenin binds to in CPC-2 cells. One possible candidate is the epidermal growth factor receptor, which has previously been shown to regulate the function of AJs [34] and to interact with β-catenin [35].

Similar to CP tissues from rats and humans, in all three CP cell lines studied, occludin was detected on immunoblots as a ~65-kDa protein. However, in both rat and human CP tissues and in Z310 cells, additional bands were detected with anti-occludin antibody. These findings are in line with previous observations that multiple isoforms of occludin are generated as a result of alternative splicing events [25]. Among these multiple isoforms, two occludin variants have been found that lacked the fourth transmembrane domain. These variants did not co-localize with ZO-1. Furthermore, a new promoter and transcription start site have been identified, adding to the structural diversity of occludin variants. The physiological significance of the presence of multiple isoforms of occludin is presently unclear.

Unlike occludin, claudin 2 protein was not found to be expressed in any of the cell lines analyzed in the present study. Although claudin 2 appears to be an important component of TJ complexes in choroidal epithelium [21,22], recent studies have suggested that claudin 2 can actually decrease the tightness of epithelial barrier by acting as a cation-selective channel [36]. At present, the functional role of claudin 2 in the choroidal epithelium is not clear, but this TJ protein may play a role in the process of CSF formation because of its cation channel properties [37]. Since claudin 2 can substantially decrease TEER of epithelial monolayers [36], its lack of expression in CP cell lines may in fact result in the enhancement of barrier properties of these *in vitro *models of BCSFB.

## Conclusion

The three CP cell lines analyzed in this study vary considerably with regard to the expression of AJ and TJ proteins, which is likely reflected by different barrier properties of these *in vitro *models of BCSFB.

## Competing interests

The author(s) declare that they have no competing interests.

## Authors' contributions

JSC cultured the cells and conducted the Western blot and immunocytochemical analyses. She also contributed to designing the experiments and writing the manuscript; CLP conducted RT-PCR analysis; ANP and CC designed the primers for PCR; AC contributed to designing the experiments and writing the manuscript. All authors read and approved the final manuscript.
